# Initial Results from a Novel Video Cholangiopancreatoscope: An International Multicenter Study

**DOI:** 10.1055/a-2965-6616

**Published:** 2026-10-07

**Authors:** David M. de Jong, Willem J. Lammers, Akin Inderson, Rogier P. Voermans, Jan Werner Poley, George J.M. Webster, Heng Chi, Mark Ellrichmann, Jörn Arne Meier, Michael Praktiknjo, Cecilia Binda, Carlo Fabbri, Marco Spadaccini, Alessandro Repici, Andrea Anderloni, Aurelio Mauro, Marco J. Bruno, Pieter Jan F. de Jonge, On behalf of the European Cholangioscopy Group

**Affiliations:** 1 Department of Gastroenterology and HepotologyErasmus MC University Medical CenterRotterdamNetherlands; 2 Department of Gastroenterology and HepatologyLeiden University Medical CenterLeidenNetherlands; 3 Department of Gastroenterology and HepatologyAmsterdam University Medical CenterAmsterdamNetherlands; 4 Cancer Centre AmsterdamAmsterdamNetherlands; 5 Department of Gastroenterology and Hepatology 199236 Maastricht University Medical Centre+MaastrichtNetherlands; 6 Department of Gastroenterology and HepatologyUniversity College London Hospitals NHS Foundation TrustLondonUnited Kingdom of Great Britain and Northern Ireland; 7 Department of Internal Medicine III – Gastroenterology and HepatologyUniversity Hospital OldenburgOldenburgGermany; 8 Department of Medicine BUniversity Hospital MünsterMünsterGermany; 9 Department of Gastroenterology and Digestive Endoscopy UnitForlì-Cesena Hospitals, AUSL RomagnaForlì-CesenaItaly; 10 Endoscopy UnitIstituto Clinico HumanitasRozannoItaly; 11 Digestive Endoscopy UnitIRCCS Humanitas Research HospitalRozzannoItaly; 12 Department of Gastroenterology and Digestive Endoscopy UnitFondazione I.R.C.C.S. Policlinico San MatteoPaviaItaly

**Keywords:** pancreatobiliary (ERCP/PTCD), cholangioscopy, pancreatoscopy, ERC topics

## Abstract

**Background** Novel 8.9-Fr and 11-Fr digital cholangiopancreatoscopes (Scivita Medical) have recently become available in Europe, but data on technical performance and safety remain limited. We report initial real-world experience with the Scivita platform.

**Methods** This international, multicenter, retrospective cohort study included consecutive patients undergoing digital single-operator cholangioscopy (dSOC) or pancreatoscopy (dSOP) between March and October 2025 at 10 European centers. The primary outcome was technical success, defined as achievement of the intended procedural goal. Secondary outcomes included intra- and post-procedural complications, classified using the AGREE classification.

**Results** In total, 77 patients (54.5% female; median age 70 years [IQR: 60–76]) were included. In 68 patients, 78 dSOC procedures achieved an overall technical success rate of 91% (71/78): choledocholithiasis 87% (33/38), biliary strictures 96% (24/25), selective cannulation 100% (2/2), evaluation of intraductal margins 50% (1/2), and migrated stent removal 100% (1/1). In all, 9 patients underwent 11 dSOP procedures, with an overall technical success rate of 63.6% (7/11): stone clearance 66.7% (4/6), intraductal papillary mucinous neoplasm assessment 50% (1/2), bridging pancreatic duct disruption 0% (0/1). No intraprocedural complications occurred. Postprocedural complications followed six dSOC procedures: cholangitis in two, mild post-ERCP pancreatitis in two, and abdominal pain without a clear substrate in two. One patient developed mild pancreatitis after dSOP.

**Conclusion** Scivita dSOC demonstrated high technical success and an excellent safety profile. Larger prospective studies are warranted to validate these findings and define the role of dSOC and dSOP of Scivita cholangiopancreatoscopy in clinical practice, while also taking into account cost-effectiveness and environmental impact.

## Introduction

Cholangioscopy and pancreatoscopy provide direct endoscopic visualization of the biliary and pancreatic ductal systems, allowing for detailed inspection, targeted tissue sampling, and a variety of therapeutic interventions. 1 Since the earliest documented attempts in the 1950s by Roca et al., 2 these techniques have evolved significantly, particularly over the past two decades. Advancements in digital imaging, scope design, and accessory technologies have propelled cholangioscopy and pancreatoscopy into mainstream clinical practice, where they are increasingly used for both diagnostic and therapeutic purposes, as described in various guidelines. 3 4 5 6 7

Direct visualization through these modalities facilitates precise visual inspection of indeterminate biliary strictures, intraductal masses, and complex stone disease. Cholangioscopy, in particular, has enabled high-resolution evaluation of mucosal and submucosal abnormalities, improving the detection of malignancy through visually targeted biopsies, with reported diagnostic accuracies somewhere around 80%. 8 In addition to diagnostics, cholangioscopy plays an essential role in therapeutic interventions such as electrohydraulic and laser lithotripsy for difficult bile duct stones, 9 selective guidewire placement in complex anatomy, 10 stricture dilation, and retrieval of migrated stents 11 or foreign bodies. 12

Pancreatoscopy, although more technically challenging, is increasingly used for the direct assessment of main pancreatic duct pathology, including intraductal papillary mucinous neoplasms (IPMNs), 13 strictures, and stones, particularly in the context of chronic pancreatitis. 14 These technologies have expanded the capabilities of endoscopic retrograde cholangiopancreatography (ERCP), transforming it from a primarily fluoroscopic procedure into a visually guided, precision intervention.

The introduction of digital single-operator cholangiopancreatoscopy systems, such as the SpyGlass DS II, has significantly improved image quality, maneuverability, and procedural efficiency. More recently, a new generation of digital cholangiopancreatoscopes has emerged. Among these are the EyeMAX, Leinzett, VedVision, Dragonfly, Seesheen CDH-1, and DRES systems, which differ in catheter diameter, working channel size, image processing, and accessory compatibility. Larger working channels may facilitate the use of larger biopsy forceps, potentially resulting in larger tissue specimens and improved diagnostic yield for indeterminate biliary strictures. 15 16 As cholangiopancreatoscopy technology continues to evolve, newer platforms are being adopted across clinical settings in Europe and worldwide. The Scivita digital cholangiopancreatoscope is one such novel platform, including multiple catheter sizes and high-resolution image processing with electronic zoom and freeze-frame capability, but data on its performance in real-world clinical settings is limited to one case report. 17 Given the growing number of available cholangiopancreatoscopy platforms and the expanding range of clinical indications, systematic evaluation of the technical performance, safety, and clinical utility of these systems is increasingly important. Such data are needed to inform evidence-based device selection and optimize the use of specific platforms for different diagnostic and therapeutic indications.

Therefore, the aim of this study was to evaluate the technical performance, safety, and clinical outcomes of the Scivita digital cholangiopancreatoscopy system across multiple centers in Europe.

## Patients and Methods

### Study Population

An international, multicenter, retrospective cohort study was conducted at 10 centers between March and October 2025. All 10 centers were members of the European Cholangioscopy group, including therapeutic endoscopists with substantial experience in dSOC (>200 lifetime procedures, outside of Scivita use). None of the endoscopists performed dSOC or dSOP before using the Scivita system. All centers obtained ethical approval from their respective local ethics committees (Erasmus MC University Medical Center, IRB approval code: MEC-2024-0617). The study followed the principles of the Declaration of Helsinki and was reported according to the STROBE guidelines. 18

Consecutive patients were identified from prospectively maintained lists of cholangiopancreatoscopy procedures. Eligible patients underwent at least an attempt of cholangioscopy and/or pancreatoscopy with the Scivita cholangiopancreatoscope, while patients aged <18 years were excluded. If cannulation with a wire failed, but the intention of the procedure was to perform dSOC or dSOP with Scivita, patients were included as well.

### Cholangioscopy/Pancreatoscopy Procedure

ERCP was performed under deep sedation, general anesthesia, or under conscious sedation with patients in the prone or supine position. The duodenoscope was advanced into the duodenum, and cannulation of the bile duct or pancreatic duct was achieved. After deep cannulation, cholangioscopy or pancreatoscopy was performed using an over-the-wire technique or by using the free hand technique. Both the 8.9-Fr and 11-Fr cholangiopancreatoscopes were used in this study (Single-use Choledocho Videoscope, Scivita Medical, China) with a working channel of 1.2 and 2.2 mm, respectively, and an bidirectional angulation range of 180° (up and down) and a 120° field of view. The choice between the 8.9-Fr and 11-Fr cholangiopancreatoscope was left to the discretion of the endoscopist based on procedural preference, duodenoscope working channel, scope availability, and clinical judgment. If considered necessary to achieve technical success, the endoscopist could switch to either the smaller or larger Scivita, or other brands of cholangiopancreatoscopes during the procedure.

Depending on the indication, the following techniques were used: electrohydraulic lithotripsy using the Autolith or Walz probe catheter for biliary and pancreatic stones, SpyBite Max for biopsies, and SpyBasket for residual stones. Biliary samples were collected and analyzed by an expert pathologist at the respective centers. Histopathologic findings were classified as benign, atypical, malignant, or inconclusive. Examples of several cholangioscopy and pancreatoscopy images are presented in Fig. 1 .

**Fig. 1:**
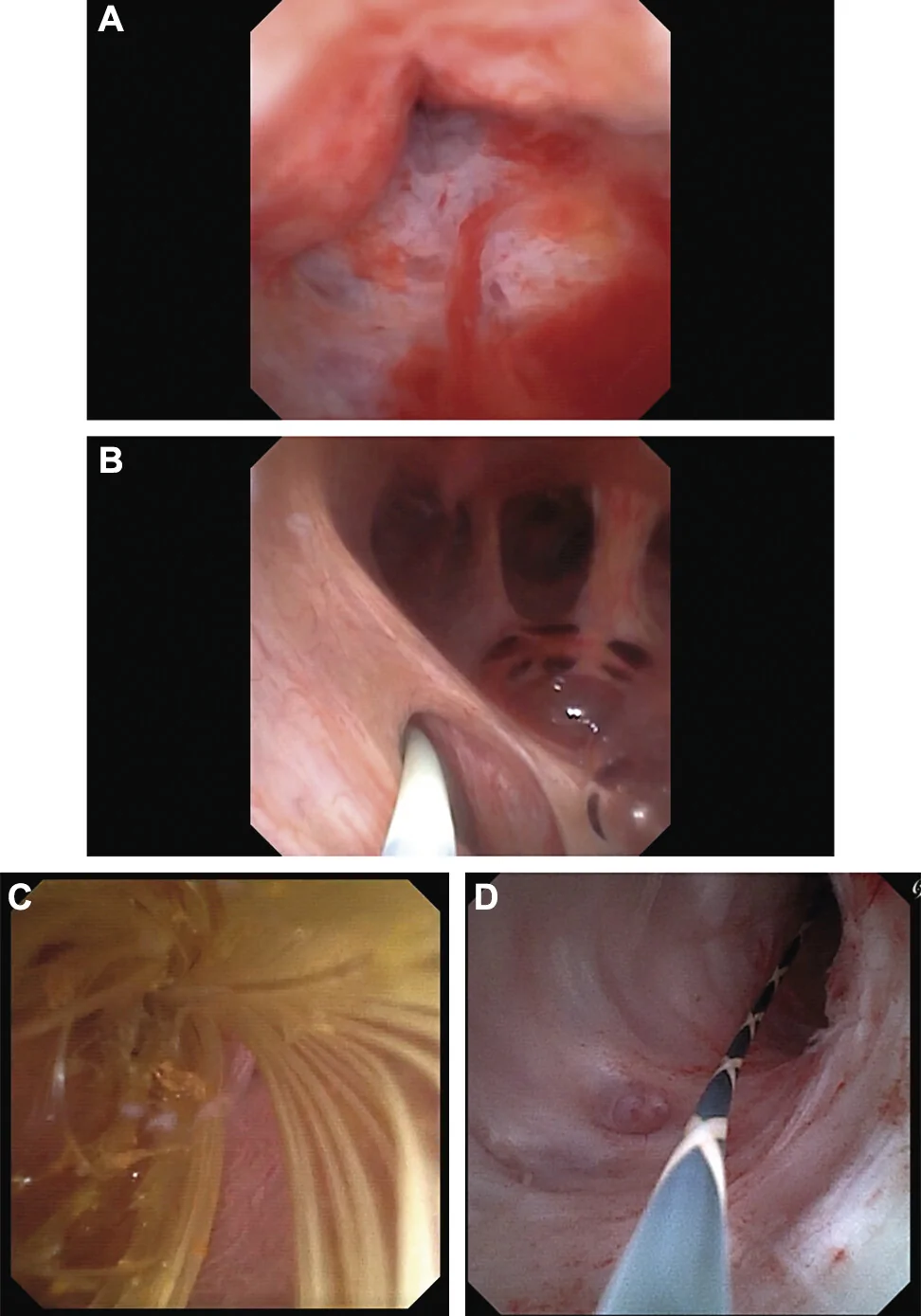
Examples of dSOC (panel A, B and C) and dSOP (panel D) ( **A** ) dSOC showing normal bile duct mucosa; ( **B** ) dSOC showing a suspicious biliary duct stricture; ( **C** ) dSOC showing a feather in the cystic duct; ( **D** ) dSOP showing normal pancreatic duct in a patient with presumed IPMN, but confirming findings more suspicious for chronic pancreatitis.

### Data Collection

Data were retrospectively extracted from electronic patient records on demographic factors (sex, age), medical history, etiology of the underlying disease, preprocedure cross-sectional imaging, as well as previous diagnostic work-up and treatments. In addition, preprocedural laboratory values were collected. Cholangioscopy and pancreatoscopy data comprised technical success of the procedure and procedural adverse events. Data collected afterward included postprocedure adverse events within one week from procedure, depending on availability from the electronic patient records, and pathologic outcomes if applicable. Patients were not contacted regarding postprocedural follow-up or pathologic confirmation in the setting of this study.

### Outcome Definition

The primary outcome was technical success of dSOC and dSOP, defined as the successful advancement of the Scivita cholangiopancreatoscope into the bile or pancreatic duct, respectively, followed by completion of the intended diagnostic or therapeutic intervention, stratified by indication. Secondary outcomes included the diagnostic yield and histopathologic concordance of dSOC-guided biopsies with visual assessment of biliary strictures, as well as procedural-related and postprocedure adverse events, graded according to the AGREE classification. 19

### Statistical Analysis

The statistical analysis included descriptive statistics using mean and standard deviation (SD) for normally distributed variables, and median and interquartile range (Q1–Q3) for non-normally distributed continuous variables. Categoric variables were described using frequencies and proportions (%). Contingency tables were constructed assessing visual diagnosis of biliary strictures compared to dSOC-guided biopsies. Benign, atypical cells or inconclusive results were considered a “negative” test outcome, whereas malignant morphology was considered a positive test outcome. All analyses were conducted using R (version 4.5.2.).

## Results

### Study Population and Baseline Characteristics

A total of 77 patients were included; 68 underwent dSOC and 9 underwent dSOP. The median patient age was 70 years (Q1–Q3: 60–76), and the majority of patients were female (54.5%). In all, 22 patients (28.6%) had prior hepatobiliary surgery. All baseline characteristics are listed in Table 1 .

**Table 1:** Baseline characteristics of the total cohort.

Variable	Total ( *n* = 77)	dSOC ( *n* = 68)	dSOP ( *n* = 9)
Age at diagnosis, median [Q1-Q3], years	70 [60–76]	72 [61–77]	64 [54–66]
Female sex – *n* (%)	42 (54.5)	39 (57.4)	3 (33.3)
WHO PS – *n* (%)*			
0–1	69 (97.2)	60 (96.8)	9 (100)
2	2 (2.8)	2 (3.2)	
ASA – *n* (%)			
I	8 (10.4)	7 (10.3)	1 (11.1)
II	36 (46.8)	30 (44.1)	6 (66.7)
III	33 (42.9)	31 (45.6)	2 (22.2)
Prior hepatobiliary surgery – *n* (%)			
Cholecystectomy	15 (19.5)	15 (22.1)	
Liver transplantation	4 (5.2)	4 (5.9)	
Pancreatoduodenectomy	1 (1.3)	1 (1.5)	
Cholecystectomy + B1 stomach resection	1 (1.3)	1 (1.5)	
Duodenum–jejunum anastomosis	1 (1.3)	1 (1.5)	
Chronic pancreatitis – *n (%)*	10 (13.0)	3 (4.4)	7 (77.8)

* = Missing in 6 patients.

IQR = Interquartile Range, WHO PS = World Health Organization Performance Status, ASA = American Society of Anesthesiologists classification.

### Cholangioscopy Procedures

A total of 68 patients underwent a total of 78 dSOC procedures. These procedures were mostly performed because of choledocholithiasis ( *n* = 46), or biliary stricture assessment ( *n* = 26). Table 2 describes the procedural indications in more detail. In the majority of procedures (64/78; 82.1%), dSOC was preplanned prior to the procedure, whereas in 14 procedures (17.9%) the endoscopist decided during the procedure to perform dSOC. Prophylactic NSAIDs were administered in 76 procedures (97.4%). One patient had an allergy for NSAIDs and in one patient there was an altered anatomy precluding any risk of pancreatitis. In 74 (94.9%) cases, procedural or postprocedural prophylactic antibiotics were administered. The majority of the procedures was performed using the 8.9 Fr scope ( *n* = 56 (71.8%)).

**Table 2:** Details of dSOC procedures.

Variable	Procedures ( *n* = 78)
Total number of patients – *n*	68
Indication of dSOC – *n* (%)	
Stones	46 (59.0)
Stricture	24 (30.8)
Stricture + stones	3 (3.8)
Selective cannulation	2 (2.6)
Migrated stent removal	1 (1.3)
IPNB assessment	1 (1.3)
Adenoma	1 (1.3)
Median procedural time, min [IQR]	76 [59 – 95]
Cholangioscope diameter – *n* (%)	
8.9 Fr	56 (71.8)
11 Fr	21 (26.9)
Both	1 (1.3)
Sedation – *n* (%)	
Conscious	7 (9.0)
Deep	59 (75.6)
General	12 (15.4)
Prophylactic antibiotics – *n* (%)*	
Procedural	65 (83.3)
Post-procedure	36 (46.2)
NSAID usage – *n* (%)	
Diclofenac	64 (82.1)
Indomethacin	12 (15.4)
Prior stent – *n* (%)	49 (62.8)
Papilla status – *n* (%)	
Prior sphincterotomy	58 (74.4)
Sphincterotomy	7 (9.0)
Sphincteroplasty	2 (2.6)
Cholangioscopy-guided biopsies – *n* (%)	20 (25.6)
Malignant	7 (35.0)
Atypical cells	4 (20.0)
Benign	8 (40.0)
Inconclusive	1 (5.0)

dSOC = digital single-operator cholangioscopy, IPNB = Intraductal Papillary Neoplasm of the Bile duct, IQR = Inter Quartile Range, Fr = French, NSAID = Nonsteroidal Anti-Inflammatory Drug.

* = Multiple can apply per procedure.

A biliary stent was in-situ in 49 procedures (62.8%). In 58 of the procedures (74.4%), sphincterotomy was already performed at a prior ERCP. A sphincterotomy was performed in 7 (9%), sphincteroplasty in 2 (2.6%), and no sphincterotomy or sphincteroplasty was performed in 11 (14.1%). One of the procedures was performed by percutaneous approach. Cholangioscope introduction into the desired duct was successful in 77 procedures (98.7%).

#### Treatment of Choledocholithiasis

Choledocholithiasis was the primary indication of the first procedure in 38 patients (55.9%). In two of these patients, stricture assessment was also intended ( Fig. 2 ). Outcomes and subsequent management were as follows:

**Fig. 2:**
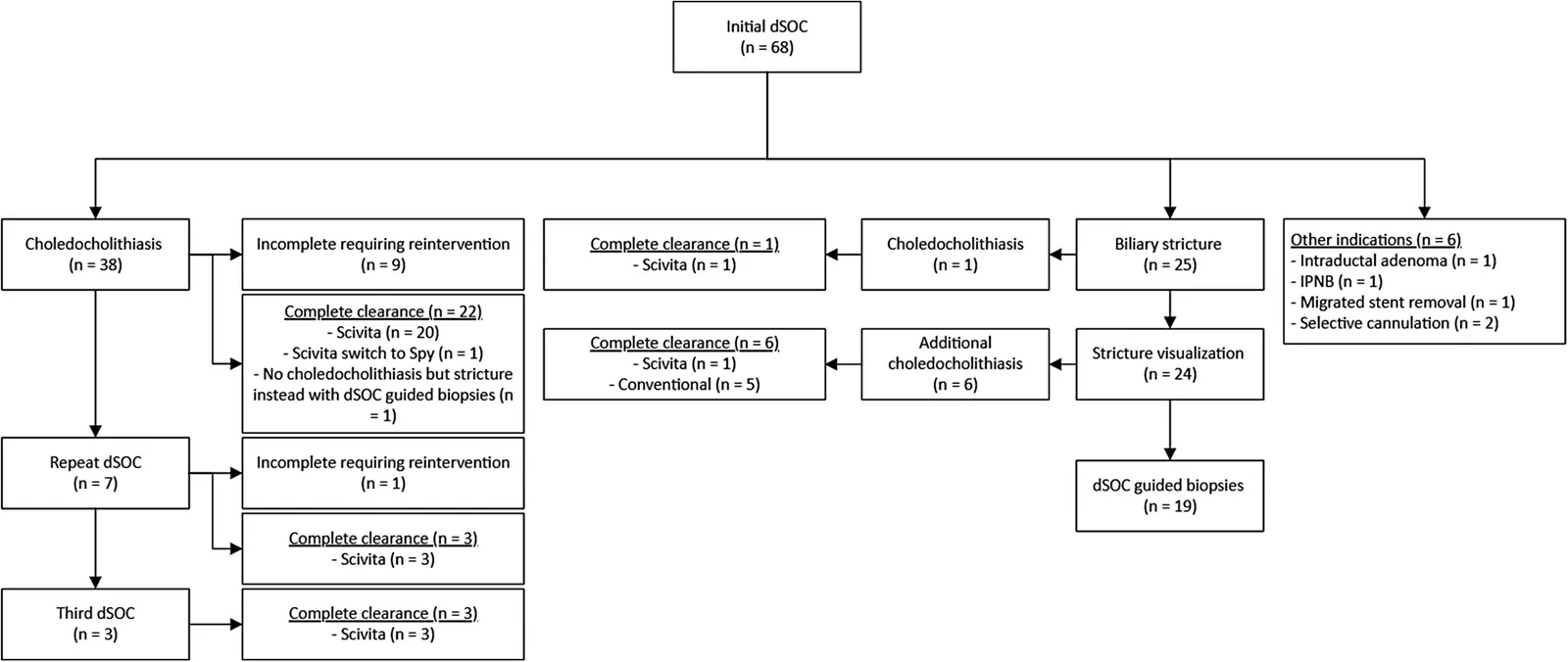
Flowchart of the included patients who underwent dSOC.

First-session ductal clearance was achieved in 22/38 patients (57.9%). A second dSOC procedure was performed in 7/38 patients (18.4%); 3 of these patients (7.9%) subsequently underwent a third procedure. Among patients undergoing repeat dSOC, complete ductal clearance was achieved at the second procedure in 3 patients and at the third procedure in 3 patients; 1 patient was planned for reintervention after stent placement. Overall ductal clearance using dSOC was achieved in 28/38 (73.7%). The remaining 10 patients were planned for reintervention. In addition, 1 patient with its initial procedure indicated for choledocholithiasis had a biliary stricture instead, of which biopsies were obtained confirming a benign stricture. In one of the patients with successful EHL of biliary stones, a small white feather was removed from the cystic duct insertion ( Fig. 1C ). Among the 48 procedures, the 8.9 Fr scope was used in 31 (64.6%), the 11 Fr scope in 16 (33.3%), and both in 1 (2.1%)

Initial dSOC failed in 5/38 patients (13.2%): switch to SpyGlass DS II in 2 patients (inadequate handling in dilated bile duct, *n* = 1; cholangioscope wheel malfunction, *n* = 1), inadequate scope position precluding stone removal in 2 patients (difficult angulation, *n* = 1; duodenal distortion, *n* = 1), and failed selective cannulation of the right posterior duct in 1 patient. Three of the failures were with the 8.9 Fr and two with the 11 Fr Table 3 describes the cross-over cases in more detail.

**Table 3:** Details of cross-over cases to other cholangioscopy platforms.

Case	Indication	Scope size used	Problem	Cross-over	Procedural success?
1	Choledocholithiasis	8.9 Fr (2x)	Device related: Cholangioscope wheel malfunction	Spyglass DS II	Incomplete removal
2	Choledocholithiasis	11 Fr	Anatomy related: Inadequate handling in dilated bile duct	Spyglass DS II	Complete removal
3	Pancreatic duct disruption	8.9 Fr	Anatomy related: Unable to visualize proximal pancreatic duct opening	Spyglass DS II	No

#### Biliary Duct Stricture Assessment

In 25 patients (36.7%), the primary indication was biliary stricture assessment: in 1 patient (4%), no stricture was visualized, but choledocholithiasis was found and removed using conventional methods using a 8.9 Fr scope. In 24 patients, a stricture was reached and visualized cholangioscopically by 8.9 Fr scope in 20 and by 11 Fr in 4. In 3 patients (12.5%), selective cannulation with the wire of the stricture itself was attempted, but only successful in 1 patient. Visual diagnosis classified 9 strictures as malignant and 15 as benign. The dSOC-guided biopsies were performed in 19 patients. Histopathologic examination confirmed malignancy in 7 of the 9 visually malignant strictures (77.8%), whereas 2 were reclassified as benign. All 11 visually benign strictures that underwent biopsy were confirmed to be benign, resulting in no false-negative visual assessments within the biopsied cohort ( Table 4 ). Findings were all concordant in procedures performed by the 11 Fr scope (5 of 5), while findings performed by the 8.9 Fr scope were concordant in 12 of the 14 ( Table 4 ), with an additional patient undergoing dSOC for choledocholithiasis, but showing a stricture, having concordant results. In the remaining 6 patients, no biopsy was attempted as the endoscopist was convinced of the benign aspect of the visualized stricture. Among the 24 patients (96%) in which a stricture was visualized, an upstream choledocholithiasis was associated in 6 patients (25%). These were treated by EHL in one patient and removed with conventional means in 5 patients.

**Table 4:** Details of dSOC-guided biopsies.

Visual diagnosis	dSOC-guided biopsy*		
	Malignant	Benign	Total
Malignant	7	2	9
Benign	0	11	11
Total	7	13	20

Procedures performed by 11 Fr were all concordant (three correct malignant diagnosis, two correct benign diagnosis), while procedures by 8.9 Fr were concordant in 13 (four correct malignant diagnosis, nine correct benign diagnosis) and discordant in 2 (visual diagnosis was malignant but dSOC-guided biopsy yielded atypical cells only).

* = Nineteen patients with the indication of dSOC being a biliary stricture, and one patient with presumed choledocholithiasis having a stricture instead.

#### Other Indications

In 1 patient undergoing 8.9 Fr dSOC, the indication was the evaluation of intraductal extension of an ampullary adenoma after endoscopic resection, confirming an intraductal adenoma growth of 1.5 centimeters. In another patient, 8.9 Fr dSOC was aimed to obtain intraductal biopsies for a lesion suspicious of intraductal papillary neoplasm of the bile duct (IPNB) causing hemobilia, but this was unsuccessful due to inadequate visualization because of active bleeding. In 1 patient, a proximally migrated straight plastic stent was removed successfully with the help of 8.9 Fr dSOC, after failure of conventional methods. In the last 2 patients using a 8.9 Fr and 11 Fr scope, respectively, dSOC was successfully used for selective cannulation and subsequent drainage of intrahepatic bile ducts.

### Pancreatoscopy Procedures

A total of 9 patients underwent ERCP with the indication to perform dSOP, resulting in a total of 11 procedures ( Table 5 and Fig. 3 ). The indications for dSOP were treatment of pancreatolithiasis in 6 patients, diagnosis of suspected IPMN in 2 patients, and treatment of suspected pancreatic duct disruption in 1 patient. Prophylactic NSAID was administered in all (100%,) and prophylactic antibiotics were administered in two (18.2%). Only one procedure was performed with the 11 Fr scope (10%), the rest with the 8.9 Fr scopes (90%).

**Table 5:** Details of dSOP procedures.

Variable	Procedures ( *n* = 11)
Total number of patients – *n*	9
Indication of dSOP – *n* (%)	
Pancreatolithiasis	8 (72.7)
MD-IPMN	2 (18.2)
Pancreatic duct disruption	1 (9.1)
Median procedural time, min [IQR]	66 [65 – 76]
Pancreatoscope diameter – *n* (%)*	
8.9 Fr	9 (90)
11 Fr	1 (10)
Sedation – *n* (%)	
Conscious	1 (9.1)
Deep	9 (81.8)
General	1 (9.1)
NSAID usage – *n* (%)	
Diclofenac	11 (100)
Prophylactic antibiotics – *n* (%)**	
Procedural	1 (9.1)
Post-procedure	1 (9.1)
Papilla status – *n* (%)	
Prior sphincterotomy	4 (36.4)
Sphincterotomy	4 (36.4)
Pre-cut	1 (9.1)

dSOP = digital single-operator pancreatoscopy, MD-IPMN = Main Duct Intraductal Papillary Mucinous Neoplasm, IQR = Inter Quartile Range, Fr = French, NSAID = Nonsteroidal Anti-Inflammatory Drug.

* = One patient had the indication to use dSOP, but failed to achieve cannulation therefore precluding dSOP use.

** = In two different procedures among 2 patients.

**Fig. 3:**
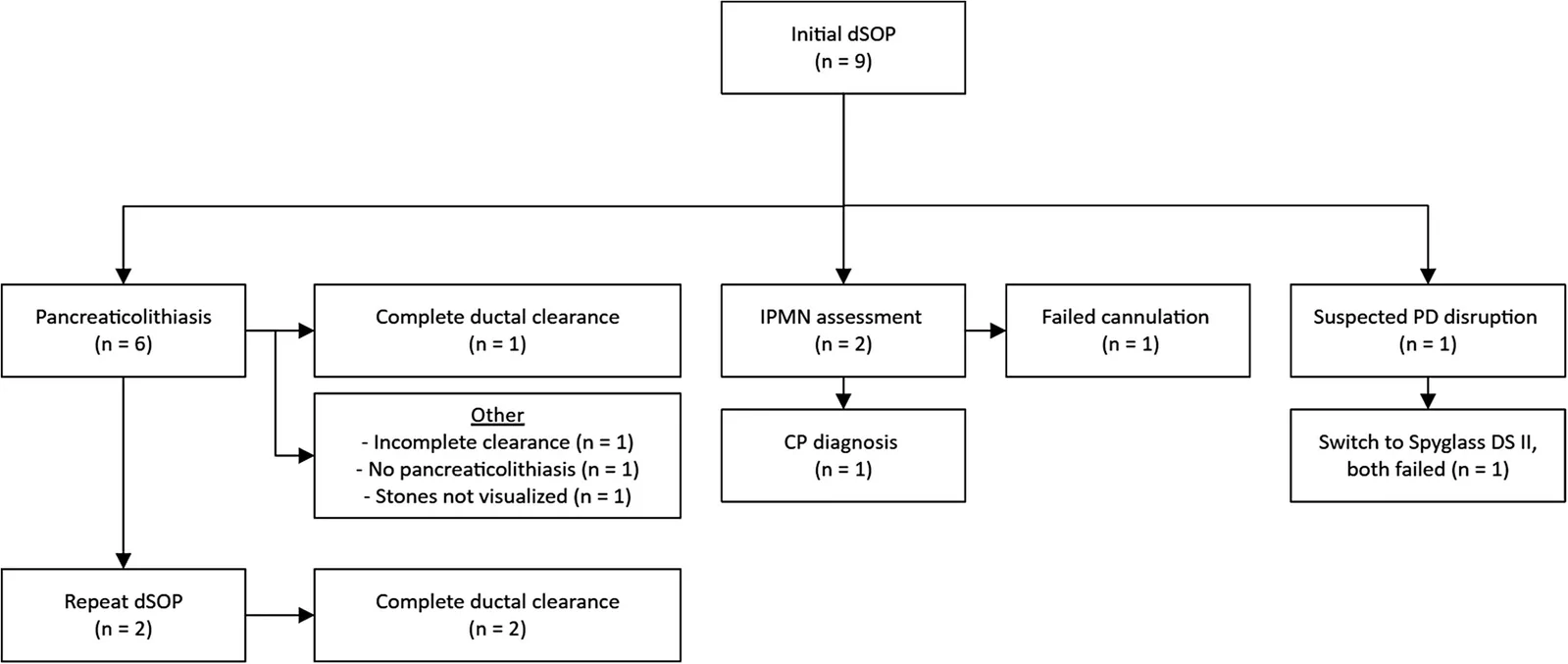
Flowchart of the included patients who underwent dSOP.

#### Treatment of Pancreatolithiasis

Among patients with pancreatolithiasis, in 1 patient deep pancreatic duct cannulation could not be achieved because of a benign stenosis, precluding stone visualization or removal. dSOP revealed mucus plugs without evidence of calculi in 1 patient. Complete ductal clearance by EHL was achieved in 1 patient, whereas another patient underwent incomplete EHL using the 11 Fr scope. In one case, technical difficulties necessitated a second procedure, during which complete EHL was successfully performed. In the sixth case, initial cannulation was unsuccessful; however, during a subsequent procedure, stone extraction was achieved using conventional methods, including removal of the remaining sheath of a Jagwire used during a previous unsuccessful endoscopic ultrasound-guided rendezvous procedure.

#### IPMN Assessment



**Video 1** Live procedure performed with the indication to assess IPMN extent, but findings were more suggestive of chronic pancreatitis


Of the 2 patients who underwent dSOP for suspected IPMN, pancreatic duct cannulation failed in 1 patient, who subsequently developed mild post-ERCP pancreatitis (AGREE II). In the other patient, in whom an IPMN of the Santorini or main duct was initially suspected, pancreatoscopic findings were more suggestive of chronic pancreatitis with a stricture and secondary ductal dilatation; therefore, no biopsies were obtained ( Video 1 ).

#### Pancreatic Duct Disruption

In the remaining patient, dSOP was performed to evaluate a suspected pancreatic duct disruption, but the procedure was unsuccessful because the transection site could not be reached. Conversion to SpyGlass DS II dSOP also failed to achieve the intended intervention. A short pigtail stent was subsequently placed for distal pancreatic duct drainage, while the proximal part of the duct remained undrained.

### Complications

No intraprocedural complications were identified. Postprocedural DSCOC complications were reported in six procedures among six patients. These included cholangitis in two cases using the 8.9 Fr scope (AGREE grade 2) despite antibiotic treatment in both, mild post-ERCP pancreatitis in two cases using the 11 Fr scope (AGREE grade 1), and abdominal pain in one case using the 8.9 Fr scope (AGREE grade 1). One patient had hypersensitivity reaction to antibiotics in one case (AGREE grade 2), but this was unrelated to the procedure. After DSOP, a complication occurred in one patient (9.1%) who developed a mild post-ERCP pancreatitis following failed cannulation for IPMN inspection with a 8.9Fr scope (AGREE II).

## Discussion

In this initial multicenter European patient series, we show that the Scivita digital cholangiopancreatoscopy system is a feasible, safe, and versatile platform for both diagnostic and therapeutic pancreaticobiliary interventions. This is the first study to evaluate the performance of this novel cholangiopancreatoscope in real-world clinical practice. Our results show high technical success rate over a broad range of indications with a safety profile comparable to currently available dSOC systems.

Regarding dSOC, the technical feasibility was excellent, with successful introduction of the cholangioscope into the CBD in 99% of procedures. This rate is in line with previously reported success rates for established platforms, such as the Spyglass DS II system. 20 An important distinguishing feature of the Scivita platform compared with the currently most widely used dSOC and dSOP system, the SpyGlass DS II (10.5 Fr with 1.2 mm working channel), is the availability of cholangioscopes with different outer diameters (8.9 Fr and 11 Fr). This provides greater flexibility in selecting the appropriate scope according to biliary anatomy and procedural requirements, for example, smaller diameter cholangiopancreatoscopes may have additional value for pancreatic interventions or difficult biliary strictures in the setting of primary sclerosing cholangitis. In addition, the larger-diameter scope offers a wider working channel, allowing the use of larger accessories and potentially improving therapeutic capabilities, particularly during lithotripsy procedures and tissue acquisition. Potential disadvantages of larger scopes is that scope advancement may be more difficult and could result in higher procedural risk due to higher papillary dilatation. In both scope sizes, the additional technology of virtual chromoendoscopy may improve visual characterization of vessels and benefit the distinction between malignant or benign strictures. Although this study was not designed to compare outcomes between different scope diameters or cholangiopancreatoscopy platforms, the availability of both 8.9-Fr and 11-Fr scopes provided procedural flexibility. Whether this translates into improved procedural or diagnostic outcomes remains to be determined in future comparative studies.

For patients with biliary stones, complete ductal clearance was achieved in a substantial proportion of patients during the initial procedure. A recent noninferiority randomized controlled trial showed that endoscopic removal of noncomplex biliary stones was 87% for fluoroscopy-free dSOC, similar to conventional ERCP. 21 This study demonstrated that dSOC may not only be a rescue technique in case of complex stones but can also serve as a potentially cost-effective primary approach in selected patients. In Europe, dSOC is predominantly offered in tertiary referral centers, to which patients are often referred after failed ERCP. It would be valuable for future studies to identify those patients in whom it is cost effective, independent of the specific cholangiopancreatoscopy system that is used, to immediately refer for dSOC and lithotripsy instead of attempting conventional ERCP first. For patients undergoing dSOC for biliary stricture assessment, the Scivita scope demonstrated promising diagnostic performance. Cholangioscopic evaluation was feasible in nearly all patients, and visual impression showed high concordance with histopathology. Although the number of patients was limited and long-term follow-up was unavailable, and most importantly lacked an established reference standard, these findings are consistent with the established value of dSOC as an adjunct for the evaluation of indeterminate biliary strictures. 4 8

With regard to dSOP, the number of included procedures was limited and therefore conclusions regarding performance should be interpreted with caution. Our findings suggest technical feasibility of dSOP using the Scivita platform in selected patients. Although successful visualization and treatment of pancreatic duct stones were achieved in several cases, the relatively small sample size and lower technical success rate compared with dSOC preclude firm conclusions regarding efficacy. 14 22 Larger prospective studies are needed to better define the role of the Scivita system in dSOP.

The safety profile of the Scivita system was favorable. No intraprocedural complications occurred. Postprocedural adverse events were observed in 7.7% of dSOC procedures and 9.1% of dSOP procedures, most events being mild or moderate according to the AGREE classification. Cholangitis and mild post-ERCP pancreatitis were the most frequent complications, consistent with the well-recognized risks associated with ERCP and cholangiopancreatoscopy, irrespective of the platform used. 22 23 Importantly, no severe adverse events, procedure-related mortality, or device-specific safety signals were identified in our cohort. Therefore, the observed adverse event profile appears to reflect the inherent risks of ERCP combined with intraductal endoscopy rather than complications attributable to the Scivita cholangiopancreatoscopy platform itself.

This study has several strengths, representing the first multicenter evaluation of the Scivita cholangiopancreatoscope, including data from 10 expert European centers and reflecting real-world clinical practice in highly experienced tertiary centers, across a broad spectrum of indications. However, several limitations should be acknowledged. First, the retrospective design limited data completeness and precluded standardized assessment of subjective parameters, such as endoscopist satisfaction. Second, although all participating centers were high-volume expert institutions; heterogeneity in local clinical practice and data collection may have influenced the results. For example, protocols regarding antibiotic prophylaxis and postprocedural follow-up varied between centers. Third, some centers contributed a limited number of procedures, mainly because they participated during the early launch phase of the device. Fourth, direct comparison with other cholangiopancreatoscopy systems was not possible. As more systems become available, head-to-head comparisons using objective clinical outcomes, ideally in randomized prospective studies, are needed. Additionally, the diagnostic performance of dSOC-guided biopsies should be interpreted with caution because not all strictures underwent histopathologic verification or adequate long-term clinical. Benign appearing lesions without a reference standard may have included unrecognized malignant strictures, potentially affecting the diagnostic accuracy of dSOC-guided biopsies. We have therefore only reported the agreement between visual diagnosis and biopsy results. Finally, cost-effectiveness and environmental impact, both increasingly important considerations for single-use endoscopic devices, were not evaluated.

In conclusion, this patient series primarily demonstrates the technical performance and safety of the Scivita platform for dSOC across a wide range of diagnostic and therapeutic indications. Experience with dSOP was more limited. Future prospective multicenter studies incorporating multiple cholangiopancreatoscopy platforms should assess technical success, diagnostic and interventional accuracy, and adverse event rate using standardized definitions. Head-to-head comparisons between different digital systems, as well as cost-effectiveness and sustainability analyses, will further guide clinical development and implementation of cholangiopancreatoscopy.

## Declaration of GenAI Use

The authors confirm that no Generative AI was used in the preparation of this manuscript.
